# Chronic Pain in Musculoskeletal Diseases: Do You Know Your Enemy?

**DOI:** 10.3390/jcm11092609

**Published:** 2022-05-06

**Authors:** Roberto Bonanni, Ida Cariati, Virginia Tancredi, Riccardo Iundusi, Elena Gasbarra, Umberto Tarantino

**Affiliations:** 1 Department of Clinical Sciences and Translational Medicine, “Tor Vergata” University of Rome, Via Montpellier 1, 00133 Rome, Italy; roberto.bonanni1288@gmail.com (R.B.); umberto.tarantino@uniroma2.it (U.T.); 2 Department of Systems Medicine, “Tor Vergata” University of Rome, Via Montpellier 1, 00133 Rome, Italy; tancredi@uniroma2.it; 3 Centre of Space Bio-Medicine, “Tor Vergata” University of Rome, Via Montpellier 1, 00133 Rome, Italy; 4 Department of Orthopaedics and Traumatology, “Policlinico Tor Vergata” Foundation, Viale Oxford 81, 00133 Rome, Italy; riccardo.iundusi@uniroma2.it (R.I.); gasbarra@med.uniroma2.it (E.G.)

**Keywords:** musculoskeletal pain, nociceptive stimulation, sensitization, musculoskeletal diseases, chronic pain, interdisciplinarity

## Abstract

Musculoskeletal pain is a condition that characterises several diseases and represents a constantly growing issue with enormous socio-economic burdens, highlighting the importance of developing treatment algorithms appropriate to the patient’s needs and effective management strategies. Indeed, the algic condition must be assessed and treated independently of the underlying pathological process since it has an extremely negative impact on the emotional and psychic aspects of the individual, leading to isolation and depression. A full understanding of the pathophysiological mechanisms involved in nociceptive stimulation and central sensitization is an important step in improving approaches to musculoskeletal pain. In this context, the bidirectional relationship between immune cells and neurons involved in nociception could represent a key point in the understanding of these mechanisms. Therefore, we provide an updated overview of the magnitude of the musculoskeletal pain problem, in terms of prevalence and costs, and summarise the role of the most important molecular players involved in the development and maintenance of pain. Finally, based on the pathophysiological mechanisms, we propose a model, called the “musculoskeletal pain cycle”, which could be a useful tool to counteract resignation to the algic condition and provide a starting point for developing a treatment algorithm for the patient with musculoskeletal pain.

## 1. Introduction

Pain is defined by the International Association for the Study of Pain (IASP) as “an unpleasant sensory and emotional experience associated with actual or potential tissue damage or described in terms of such damage”. In addition to this definition, the IASP carries some notes defining pain as a personal experience that can be influenced by biological, psychological, and social factors [1]. Although this definition describes the concept of pain, it does not provide information on the type of pain an individual may experience, explaining the need for the subsequent introduction of further descriptors of pain to more accurately define the mechanisms involved in its development.

An important definition, which has the advantage of being clear and operational, is that of chronic pain, which is pain that recurs or persists for more than three months. This can be distinguished into chronic primary pain, i.e., pain in one or more anatomical sites that persists or recurs for more than three months and is associated with emotional distress and functional disability; chronic cancer pain, i.e., due to a malignancy and defined according to site; post-surgical pain; orofacial pain; musculoskeletal pain [2,3]. Recently, special attention has also been paid to neuropathic pain, defined by the International Classification of Diseases 11th Revision (ICD-11) as a lesion or disease of the somatosensory nervous system that may result in function loss and increased sensitivity to pain and spontaneous pain [4]. However, in a wide variety of cases, pain may be experienced due to stimulation of a nociceptor, i.e., a high threshold receptor in the somatosensory nervous system capable of transducing and encoding noxious stimuli, rather than due to injury to the somatosensory nervous system. In this case, we speak of nociceptive pain defined as pain that results from actual or threatened damage to non-neural tissue and is due to nociceptor activation [5].

However, the classification into neuropathic pain and nociceptive pain is not sufficient to explain some pain conditions, such as non-specific back pain, fibromyalgia, and complex regional pain syndrome type I (CPRS-1). Therefore, the introduction of a third pain descriptor, nociplastic pain, was necessary. This was defined as pain resulting from altered nociception, without clear evidence of tissue damage, which could justify nociceptor activation or evidence of a disease of the somatosensory nervous system causing the pain. Nociplastic pain is characterised by allodynia, i.e., pain induced by a normally non-painful stimulus, hyperalgesia, i.e., increased pain perception induced by a painful stimulus, and reversibility [6]. Importantly, the distinction between nociceptive and nociplastic pain is not always well-defined as patients may have a combination of both types of pain [7].

Pain is an important symptom of joint and musculoskeletal disorders that contributes to loss of function. As such, it is indicative of the severity and activity of the underlying disease condition and can be used as a prognostic and/or therapeutic indicator of the disease state [8]. However, it should not be forgotten that pain is a well-defined condition that needs to be treated independently of the underlying disease process. Unfortunately, studying pain is by no means straightforward and involves several issues. Firstly, the onset of pain is not always defined with certainty because the patient struggles to place the onset of the symptom in time [9]. Secondly, although there are several scales to measure intensity, such as the visual analogue scale (VAS), the numeric pain rating scale (NPRS) and the verbal rating scale (VRS), the experience of pain is very subjective and is influenced by social, psychological, and genetic factors [8]. Finally, a specific disorder is often referred to by different terms, making it difficult to compare data from the literature and increasing the difficulty of studying the underlying mechanisms.

Based on this evidence, the aim of our narrative review was to (i) summarise current knowledge on the prevalence and burden of musculoskeletal pain, (ii) provide an overview of the underlying pathophysiological mechanisms, and (iii) highlight how a better understanding of these processes may reduce the risk of chronicity and improve the clinical outcome of patients with chronic pain associated with musculoskeletal disorders.

## 2. Literature Search Strategy

For this narrative review, 159 papers were selected from the Medline bibliographic database, published between 1945 (starting date) and 2022. Papers were included on the impact of chronic pain on patients with musculoskeletal disorders. The search strategy was based on the use and/or combination of the following keywords: “chronic pain”; “musculoskeletal pain”; “nociceptive stimulation”; “neuroplasticity”; “sensitization”; “nociceptive neuropeptides”; “musculoskeletal diseases”; “osteoarthritis”; “osteoporosis”; “CRPS-1”; “fibromyalgia”. The search process was performed on a worldwide basis, without excluding specific geographic areas or different ethnic groups. Language and species filters were applied to the results list to eliminate non-English articles.

## 3. Chronic Musculoskeletal Pain: General Aspects

In the 10th edition of the International Classification of Diseases (ICD-10), musculoskeletal pain was defined as chronic pain arising from musculoskeletal structures such as bones and joints. However, this definition was provided by considering only musculoskeletal pathologies and tissue damage responsible for the pain development, without reference to underlying mechanisms [10]. ICD-11 subsequently integrated the biomedical, psychological, and social aspects involved in the complex experience of chronic musculoskeletal pain, distinguishing between primary and secondary pain.

Primary chronic musculoskeletal pain is a well-defined condition that identifies disorders such as fibromyalgia, CPRS-1, and non-specific lower back pain. In contrast, secondary chronic musculoskeletal pain is a symptom dependent on a condition classified elsewhere, caused by persistent nociceptive stimulation in musculoskeletal structures. In addition, it may be caused by (i) persistent inflammation, due to active or latent infection, deposition of crystals generating acute inflammation, or autoimmune and autoinflammatory diseases; (ii) structural changes, such as osteoarthritis, spondylosis, or musculoskeletal injuries; (iii) diseases of the nervous system, such as Parkinson’s disease, multiple sclerosis, and peripheral neurological diseases [1,11].

### 3.1. Prevalence

Evidence has focused on the impact and prevalence of chronic musculoskeletal pain in different populations. In this regard, Breivik et al. conducted a large-scale telephone survey to investigate the prevalence, severity, and impact of chronic pain in 15 European countries and Israel. Of a total of 46,394 participants, 19% complained of moderate to severe chronic pain in the previous six months and several times during the last week, resulting in impaired quality of social and working life. Particularly, almost half of the patients suffered from back pain and more than 40% had joint pain, most frequently in the knee. However, very few patients were managed by pain specialists and almost half received inadequate pain management, highlighting that chronic pain represents a serious health problem that is not always well treated [12]. In 2011, Nakamura and colleagues conducted a cross-sectional study to collect epidemiological data on chronic musculoskeletal pain in the Japanese population by administering a postal questionnaire asking about musculoskeletal pain severity, location, duration, and treatment history. Of a total of 11,507 participants, 15.4% had chronic musculoskeletal pain, with a significantly higher prevalence in women. The most affected age group was between 30 and 50 years, and the most common sites of musculoskeletal pain were the lower back, neck, shoulder, and knee [13]. Subsequently, Casser and Schaible reported that, in Germany, 22% of women and 15% of men suffered daily from lower back pain for at least three months, with a higher prevalence in individuals aged between 50 and 59 years [14]. In agreement, Meucci and colleagues observed that the prevalence of chronic lower back pain follows a linear increase from the third decade of life until the age of 60 years and is more prevalent in the female population [15].

In 2019, Latina et al. conducted an observational, multicentre, cross-sectional study to investigate the clinical characteristics of 1606 patients who visited pain management clinics in the Lazio region of Italy. Of these, 54% reported severe pain, with a significant female prevalence. Furthermore, the pain was mainly musculoskeletal (45%), but also mixed (34%) and neuropathic (21%). In 60% of the cases, the pain was continuous, and in 20% it lasted for more than 48 months, highlighting that in Italy more than a quarter of the population complains of chronic pain [16]. More recently, Øverås and colleagues conducted a systematic review to investigate the prevalence of musculoskeletal pain together with persistent back pain (>4 weeks) in high-income countries in Europe, the United States, and Japan. In a total of 34,492 individuals, persistent back pain was often associated with axial (20–60%), extremity (6–50%), or multi-site (10–89%) musculoskeletal pain and was more prevalent in the female population. Although methods of assessing concomitant pain varied significantly between the selected studies, persistence of back pain was suggested as a factor closely associated with multisite pain, chronic pain, and chronic widespread pain [17].

Taken together, the evidence shows that the female population is more affected by pain disorders, and that reports of pain increase with age.

### 3.2. Socio-Economics Costs

Musculoskeletal disorders are the leading cause of disability worldwide and represent a growing problem due to the ageing population [18]. For example, back pain, which has been reported to be the leading cause of disability in both high-income and developing countries, results in an increased fear of pain in the elderly population. As a result, older people reduce activity levels to avoid movements that cause pain, promoting sedentariness and social isolation [19].

Musculoskeletal disorders affect many other aspects of life and are responsible for reduced physical activity, increased frailty, depression, and cognitive impairment, leading to huge social and economic costs. In fact, the combined analysis of three international cross-sectional surveys showed that musculoskeletal pain has a negative impact on emotional well-being and quality of life, suggesting the need to adopt a biopsychosocial approach to pain that allows us to assess not only the intensity of pain, but also the extent to which it negatively affects an individual’s life [20].

Estimating the overall economic costs of musculoskeletal disorders is by no means easy. Indeed, to the high direct costs of care associated with treatment must be added the indirect costs due to reduced work productivity, lost wages, days of disability and lost working hours. For example, in the United Kingdom, with a population of 70 million people, back pain has been estimated to have direct costs of approximately GBP 1.6 billion and indirect costs of approximately GBP 10 billion, while for the treatment of osteoarthritis and rheumatoid arthritis a total estimate of approximately GBP 10.2 billion was reported in 2017 [21].

Of note is that pain related to musculoskeletal disorders is strongly associated with work, accounting for 62% of all occupational diseases. In 2013, in North America, the economic burden of work-associated musculoskeletal pain was estimated to cost approximately USD 25 billion annually; while in Japan, the total annual cost of medical expenses associated with lower back pain was estimated to be approximately USD 746.7 million [22]. Finally, in 2019, in the United States, back pain and osteoarthritis, the costliest and most prevalent musculoskeletal disorders (33.9% and 10.4%), were associated with total medical expenditures of USD 365 billion and USD 460 billion, respectively, confirming the significant impact of these painful conditions on the individual and the community in health, social, and economic terms [23].

### 3.3. Risk Factors

**Figure 1:**
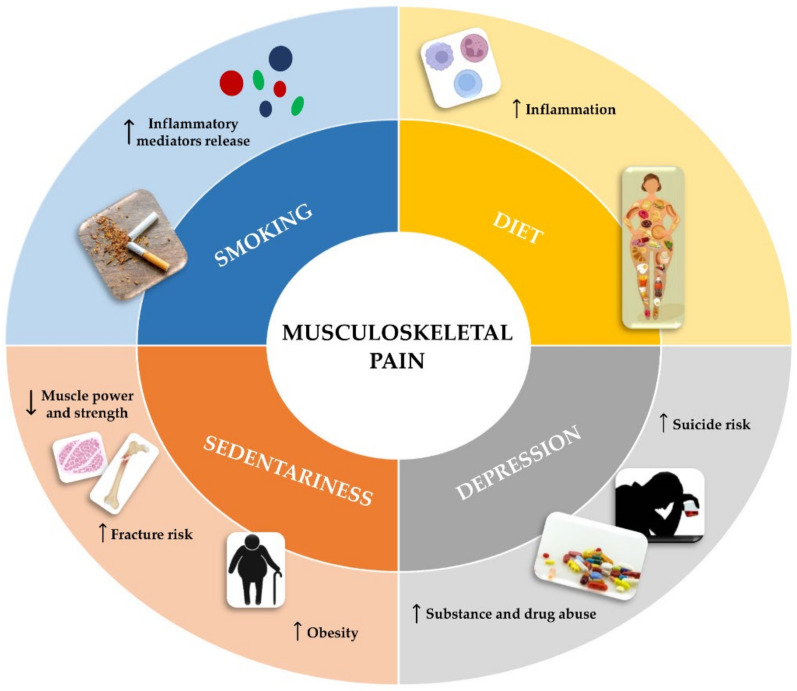
Main risk factors associated with musculoskeletal pain. Some risk factors may promote the musculoskeletal pain onset directly or by influencing lifestyle and work activity. Smoking and a diet high in animal protein and fat are factors that amplify the chronic pain associated with musculoskeletal disorders by stimulating immune cells to release inflammatory mediators. Depression, which can lead to substance and drug abuse and suicide risk increase, is also associated with musculoskeletal pain onset. Finally, a sedentary lifestyle, correlated with reduced muscle mass and strength, fracture risk increase and obesity, is among the main factors promoting chronic pain development.

Musculoskeletal pain has been suggested to be often influenced by lifestyle and work activity, as some risk factors predispose to pain either because of the direct impact they have on the musculoskeletal system or because they influence lifestyle by promoting the onset of pain [24,25]. Figure 1 shows some of the main risk factors, such as smoking, diet, depression, and sedentary lifestyle, which are widely known to be associated with musculoskeletal pain.

#### 3.3.1. Smoking

The correlation between smoking and musculoskeletal disorders is not new, as smokers are known to be characterised by lower bone mineral content (BMC) and consequently higher susceptibility to osteoporosis [26]. Several epidemiological investigations of postmenopausal women have confirmed a correlation between smoking and bone loss, showing that women who smoke experience cortical bone loss faster than non-smoking women [27].

The impact of smoking on bone health may be due to the action of nicotine, which is known to affect bone metabolism in a biphasic manner, exerting a stimulatory effect at low doses and an inhibitory effect at higher doses [28]. The deleterious effects of nicotine on bone tissue also result in increased collagen degradation and reduced blood and oxygen supply, conditions that predispose to lower back pain. In addition, smoking has been reported to have anti-estrogenic activity, as reduced estrogen levels and early menopause have been found in smokers compared to non-smokers [29]. Predisposition to chronic pain may also depend on nicotine action on the immune system, as smoking has been reported to stimulate the release of inflammatory mediators by T lymphocytes, exacerbating the musculoskeletal conditions underlying the onset of pain [30]. Interestingly, a correlation between smoking and pain intensity has been found, as evidenced by the higher intensity of chronic pain, reduced function, lower sleep quality, and worse mood found in smokers compared to non-smokers. Finally, smoking also profoundly influences healing processes and improvement over time, thus, assuming prognostic importance [31]. Interestingly, Yamada et al. found a correlation between smoking, musculoskeletal pain, and occupational disability in Japanese workers, suggesting that the influence of smoking on pain intensity may be due to an increase in certain biomarkers of inflammation, such as the C-reactive protein (CRP), which causes greater sensitivity to pain. In addition, the authors reported smoking workers were more likely to consume more than 45 g/day of alcohol, have shorter sleep duration (<5 h), and less likely to exercise regularly, confirming the association between smoking and occupational disability due to pain [32].

Overall, smoking cessation represents an important therapeutic goal in people with chronic pain, highlighting the need to intervene on this risk factor both to reduce its impact on bone tissue and to reduce its effects on inflammation, conditions that promote the onset of pain. At the same time, smoking cessation could positively influence healing processes and, more generally, contribute to improved quality of life.

#### 3.3.2. Diet

Some scientific evidence suggests an important role of diet in musculoskeletal disorders and pain onset. Particularly, diets high in animal protein and fat have been associated with inflammation and chronic pain, conditions marked by the serum CRP presence. In fact, an increase in CRP, also known as acute phase protein, is typical of pain-associated conditions, such as chronic inflammatory lower back pain, leading to a decrease in the pain threshold, function, and increased weakness. In contrast, a predominantly plant-based diet and an improved lifestyle in general can significantly influence CRP levels and exert significant benefits on musculoskeletal and pain conditions [33,34]. In this regard, Towery and colleagues investigated the effects of feeding a predominantly plant-based diet rich in cereals, fruit, vegetables, dairy products, and eggs to fourteen subjects with chronic musculoskeletal pain for eight weeks and reported significant reductions in pain scores, a slight decrease in weight, and improved quality of life as the main benefits [35].

The close relationship between diet and musculoskeletal pain was also highlighted by Elma et al., suggesting that plant-based diets may have pain-relieving effects on chronic musculoskeletal pain [36]. Interestingly, patients with fibromyalgia, who may show deficiencies in carbohydrates, protein, lipids, folate, selenium, zinc, and vitamins A, E and K, reported significantly lower VAS pain scores after eight weeks of a diet low in fermentable oligo- and monosaccharides and polyols (FODMAP). Similarly, pain severity in patients with chronic osteoarthritis was positively correlated with fat and sugar intake, suggesting the use of a weight-loss diet to reduce pain severity [36].

The effect of an adequate diet on musculoskeletal pain may lie in improving the inflammatory state. In this regard, Mendonça and colleagues showed that the adoption of a vegan or Mediterranean diet, as well as specific foods such as blueberry or fish oil, significantly reduced musculoskeletal pain. In addition, a correlation between diet, musculoskeletal pain, and inflammatory status has been found, suggesting that appropriate nutritional intervention could alleviate musculoskeletal pain, especially in patients with osteoarthritis, by reducing levels of inflammatory markers, such as interleukin-6 (IL-6), interleukin-1β (IL-1β), and tumor necrosis factor-α (TNF-α) [37].

In conclusion, evidence confirms diet as one of the most profound influences on quality of life and, more specifically, on musculoskeletal pain, with benefits possibly arising from weight loss or reduction in inflammatory markers, or both [38]. Although poor diet should be considered a predictive, perpetuating, and underlying factor in musculoskeletal pain and its chronicity, further studies are needed to clarify the molecular mechanisms involved in this relationship [39,40].

#### 3.3.3. Depression

Pain is not only a sensory experience, but also an emotional experience that can have extremely different impacts on people. According to some evidence, between 30 and 60% of people with chronic pain also suffer from depression. Furthermore, the existence of a bidirectional relationship between depression and chronic pain has been reported, as pain is a strong predictor of depression severity and vice versa [41].

The depressive state, which can lead to substance and drug abuse, can increase the risk of suicide. In this regard, Dreyer et al. hypothesised that the increased suicide rates observed in a Danish cohort of individuals with fibromyalgia could be due to increased rates of depression and anxiety, although a direct correlation has not been established and the patients who died by suicide did not have a pre-existing psychiatric diagnosis at the time of the fibromyalgia diagnosis [42]. In agreement, Baskan and colleagues observed greater musculoskeletal pain and moderate or vigorous levels of depression in unemployed women than in employed women who showed mild levels of depression, probably due to their concentration at work and their economic independence [43].

Undoubtedly, the association between musculoskeletal pain and depression further worsens an individual’s health condition and drastically reduces the quality of life, increasing the degree of disability of the patient. In this regard, Aragonès and colleagues conducted a randomised controlled trial (RCT) to evaluate the effectiveness of an integrated intervention programme in the management of patients with musculoskeletal pain and depressive symptoms. Although the severity of depression was found to be significantly improved, no progress with pain status was observed, suggesting the need to develop further complex clinical approaches for the concomitant treatment of depressive symptoms and chronic musculoskeletal pain [44].

#### 3.3.4. Sedentariness

Sedentariness combined with low physical activity is among the main risk factors for musculoskeletal pain. In fact, exercise is known to be an important preventive health strategy, and inadequate activity levels are associated with the development of a wide variety of diseases [45]. On the contrary, evidence has shown that more physically active individuals are less likely to develop chronic pain [46,47].

A sedentary lifestyle is also considered a predictor of non-specific lower back pain (nsLBP). Particularly, sedentariness has been observed to reduce muscle power and strength and decrease the ability of the spinal disc to maintain an optimal water concentration, suggesting the involvement of these factors in the development of degenerative lesions and increased risk of spinal disc herniation [48].

It is noteworthy that chronic musculoskeletal pain is a limiting factor for physical activity, so individuals experiencing it are encouraged to adopt a sedentary lifestyle. In this regard, Stubbs et al. observed that elderly people with musculoskeletal pain spent 3.5 h more of their time as sedentary than their counterparts without pain, suggesting a role of musculoskeletal pain in increasing kinesiophobia, i.e., fear of movement and fear of falling, and consequently adopting sedentary behaviour [49].

Finally, sedentariness, together with poor diet, are important risk factors for obesity development. Although this is a multifactorial and extremely complex problem, it is associated with a wide group of disorders and conditions, including musculoskeletal pain [50]. Obesity has now reached pandemic proportions, which significantly increases the risk of developing musculoskeletal pain. Information regarding the association between obesity and musculoskeletal pain is currently scarce; however, understanding the origin of this relationship is critical to developing appropriate treatment strategies and reducing its dramatic impact on quality of life [51].

## 4. Pathophysiology of Chronic Musculoskeletal Pain: Mechanisms of Central and Peripheral Sensitization

The perception of pain begins with the activation of peripheral nociceptors that transduce and encode noxious stimuli. The cells capable of encoding noxious stimuli, nociceptive neurons, originate from neural crest stem cells that migrate dorsally from the neural tube and develop late during neurogenesis. Their cell bodies are in the dorsal root ganglion (DRG) and give rise to an axon characterised by a peripheral branch that innervates the target tissue, and a central termination that enters the central nervous system (CNS), forming synapses with second-order nociceptive neurons [52].

Importantly, differences between the sensory fibres innervating the various target tissues have been reported. Specifically, bones and joints, as well as ligaments, capsules, menisci, and muscles are mainly innervated by Aδ fibres, which are finely myelinated sensory nerve fibres with conduction velocities between 2 and 30 m/s; while TrkA + C fibres, which are unmyelinated nerve fibres with conduction velocities <2 m/s, are known to express the tropomyosin receptor kinase A (TrkA) and to release the calcitonin gene-related peptide (CGRP). These nerve fibres, together with sympathetic adrenergic and cholinergic nerve fibres, contribute to pain transmission [53].

Defining how these nociceptive neurons are stimulated and respond to stimuli is a key step in understanding the development and chronicity of pain in diseases of the musculoskeletal system.

### 4.1. Nociceptive Stimulation

**Figure 2:**
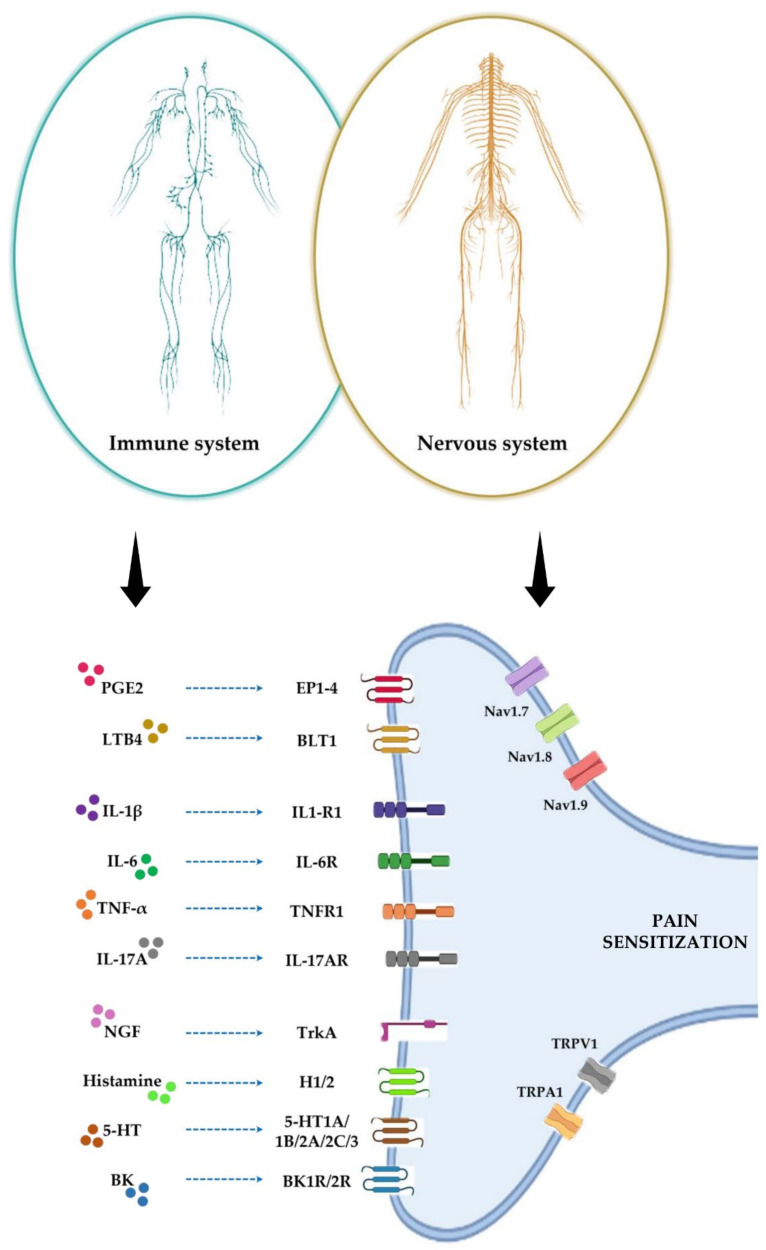
The pain development process occurs through a bidirectional crosstalk between immune cells and nociceptive neurons. During inflammation, cells of the immune system secrete a wide variety of molecular mediators responsible for nociceptive stimulation. These include lipid mediators, such as prostaglandin E2 (PGE2) and leukotriene B4 (LTB4); cytokines, such as interleukin-1 beta (IL1β), interleukin-6 (IL-6), tumour necrosis factor-α (TNFα), and interleukin-17A (IL-17A); growth factors and neurotransmitters, such as nerve growth factor (NGF), histamine, serotonin (5-HT), and bradykinin (BK). All these mediators interact with their receptors on peripheral nerve terminals, causing sensitization to pain.

Peripheral nociceptive endings express ligand-dependent and voltage-dependent ion channels, such as transient receptor potential cation channel subfamily V member 1 (TRPV1), transient receptor potential cation channel subfamily A member 1 (TRPA1), NAv1.7, NAv1.8, and NAv1.9, which are activated in response to a chemical signal and propagate it within milliseconds [54]. The chemical signal that leads to the activation of these ion channels is released by immune cells residing in peripheral tissues. These cells promote the nociceptive neurons stimulation through the release of chemical mediators, including lipids, cytokines, growth factors, and neurotransmitters, resulting in pain sensitivity, while nociceptive neurons respond by releasing neuropeptides and neurotransmitters that act on immune cells. This phenomenon results in a bidirectional crosstalk between immune cells and nociceptive neurons responsible for pain development processes [55] (Figure 2).

#### 4.1.1. Lipids

In tissue damage or during an inflammatory process, prostaglandin E2 (PGE2) stimulates nociceptive neurons through interaction with EP1-4 receptors. Among these, EP4 plays an important role in the pain development, as PGE2/EP4 signalling together with TRPV1 signalling have been suggested to promote pain chronicity. Furthermore, PGE2 expression could chronically increase the expression of other pain mediators and TRPV1 in DRG neurons, promoting the transition from acute to chronic pain [56]. In this regard, a daily injection of PGE2 is known to induce a hypernociceptive state in rats that persists even after administration cessation. This response was associated with activation of the nuclear factor kappa-light-chain-enhancer of the activated B cells (NF-kB) pathway mediated by protein kinase A (PKA) and protein kinase C (PKC) in DRG neurons, as demonstrated by the significant reduction in the period of hypernociception observed after treatment with an inhibitor of NF-kB activation [57].

The TRPV1 ion channel is also activated in response to other lipid molecules, including bioactive lysophosphatidic acid (LPA), known to evoke algesia responses in control mice that were not observed in Trpv1-null mice [58]. Furthermore, sphingosine-1-phosphate (S1P) has been suggested to act by enhancing TRPV1 activity through the involvement of a G-protein-coupled receptor expressed on sensory neurons [59]. Leukotriene B4 (LTB4) is also an important lipid mediator of pain and exerts its action through high-affinity binding to the leukotriene B4 receptor 1 (BLT1). The importance of LTB4-BLT1 signalling in pain development has been demonstrated in BLT1 knockout mouse models, characterised by a marked reduction in the nociceptive response and a significant reduction in edema formation and neutrophil infiltration, showing that the LTB4-BLT1 axis plays a crucial role in inflammatory processes and in the onset of acute and persistent pain [60].

Thus, scientific evidence suggests that certain lipid mediators, released during inflammatory processes or in the context of tissue damage, may promote the activation of the TRPV1 ion channel and subsequent depolarization of nociceptive neurons, inducing pain sensitivity.

#### 4.1.2. Cytokines

Cytokines released by immune cells during inflammation play a critical role in pain sensitivity. Interleukin-1 beta (IL-1β) is abundantly expressed during diseases such as rheumatoid arthritis and osteoarthritis. DRG nociceptors express interleukin-1 receptor type 1 (IL1R1), so IL-1β signalling on these neurons could be responsible for the onset and sensitivity to pain. Indeed, deletion of the IL1R1 gene in nociceptors expressing the TRPV1 ion channel prevents the allodynia development but does not counteract disease progression. Interestingly, restoration of IL1R1 expression has been shown to induce typical pain behaviours and transient allodynia, without further influencing the joint damage that characterises osteoarthritis [61]. Furthermore, activation of adenosine monophosphate-activated protein kinase (AMPK) exerts an analgesic effect in mice undergoing inflammatory pain by reducing IL-1β levels and suppressing translocation of the p65 subunit of NF-kB into the nucleus [62].

Interleukin-6 (IL-6) also promotes the stimulation of nociceptors by increasing pain sensitivity. IL-6 signalling to target cells requires interaction with its receptor and the presence of a glycoprotein called gp130. Specifically, interaction of IL-6 with its receptor (IL-6R) leads to the dimerization of gp130 and the subsequent activation of IL-6 signalling, which involves janus kinase (JAK) and, further downstream, proteins such as Ras and Raf. However, IL-6 can act through interaction with a membrane receptor, activating “cis” signalling, or through a soluble receptor, activating “trans” signalling, which is known to promote the activation of primary afferent sensory neurons and pain sensitivity [63,64]. The ablation of small diameter DRG neurons expressing gp130 Nav1.8 reduced thermal and mechanical hypersensitivity in mouse models of inflammatory pain (SNS-gp130-/-). In addition, a significant reduction in TRPA1 expression was observed in these mice, further confirming the involvement of IL-6 signalling in pain development processes [65].

Peripheral inflammation is characterised by increased expression of cyclin-dependent kinase 5 (CDK5) and its activator p35 in nociceptive neurons. TNF-α, released during tissue damage or inflammation, up-regulates p35 expression and, consequently, promotes Cdk5 activity, which induces TRPV1 phosphorylation increasing pain sensitivity and reactive oxygen species (ROS) production [66]. The role of TNF-α in increasing pain sensitivity depends on its ability to favour the release of other inflammatory cytokines, such as IL-6 and IL-1β, by promoting the production of PGE2 and other mediators that sensitize nociceptive neurons and induce hyperalgesia. However, although stopping TNF-α synthesis is not sufficient to interrupt the proinflammatory cytokines production, it is sufficient to prevent hyperalgesia in rats treated with carrageenan, a set of marine polysaccharides isolated from algae, confirming the TNF-α signalling involvement in the pain susceptibility of nociceptors [67].

Arthritic pain is also influenced by the interleukin-17A (IL-17A) production by T-helper 17 (Th17) lymphocytes, as demonstrated by the increased sensitization of nociceptive C-fibres to mechanical stimuli found even after a single injection into the knee joint of rats responsible for the TNF-α and IL-6 signalling disruption. Therefore, since DRG neurons contain the IL-17A receptor (IL-17AR), signalling triggered by cytokine binding to its receptor could play a central role in the mechanical hyperalgesia evoked by inflammation [68]. Furthermore, in a mouse model of antigen-induced arthritis, IL-17A knockout led to a significant reduction in mechanical hyperalgesia compared to wild-type mice but did not affect disease severity or course. Thus, IL-17A does not play a central role in the pathogenesis of antigen-induced arthritis but certainly contributes decisively to the development of inflammatory pain [69].

#### 4.1.3. Growth Factors and Neurotransmitters

Nerve growth factor (NGF) is produced and released by immune cells during inflammation or tissue damage and binds its receptor TrkA on nociceptive neurons, contributing to increased susceptibility to pain. Indeed, many inflammatory and autoimmune conditions, such as chronic arthritis, are associated with increased levels of NGF, which modulates the function of sensory nociceptors, promoting the hyperalgesia typical of inflammatory phenomena [70]. In humans, a single administration of NGF is sufficient to induce persistent pain that lasts for weeks, probably because the activation of TrkA promotes further NGF release, along with other mediators of inflammation, suggesting an important role for this neurotrophin not only in the development of hyperalgesia and allodynia, but also in the mechanisms that promote pain chronicity. Interestingly, NGF treatment is known to induce in rats an increase in TRPV1 activity and expression in DRG neurons, as well as an increase in arachidonic acid-derived TRPV1 agonists, suggesting that the NGF-induced increase in the nociceptive state may involve lipid-mediated TRPV1 activation [71].

Histamine is also known to promote Nav1.8 expression in a culture of DRG neurons, probably through interaction with the H2 receptor since the increased expression of Nav 1.8 is counteracted by the H2 receptor antagonist, cimetidine. Furthermore, the peri-sciatic administration of histamine in rats induced the up-regulation of Nav 1.8 in DGR L4/L5 neurons and attributable behaviour to mechanical allodynia and thermal hyperalgesia. However, there is evidence to support the H1 receptor involvement in the stimulation and sensitization of nociceptive fibres by histamine [72].

Serotonin (5-HT) modulates nociceptive transmission through the involvement of various receptor subtypes, which may have pro- or anti-nociceptive effects. For example, the 5-HT1A receptor inhibits nociceptive transmission at the presynaptic level, while the 5-HT1B receptor may act as a nociceptive facilitator. In addition, 5-HT2A receptor antagonists may have an important analgesic action, suggesting the involvement of this receptor in nociceptive transmission. The use of 5-HT3 receptor agonists also had analgesic effects and, similarly, the administration of 5-HT2C receptor agonists had anti-allodynic effects. Thus, the role of 5-HT in hyperalgesia depends on both the receptor involved and its distribution and may have the effect of inhibiting, exciting, or maintaining the nociceptive response [73].

Finally, bradykinin (BK) acts as a mediator of a wide variety of physiological and pathophysiological responses, including pain and inflammation. This neurotransmitter excites DRG neurons through interaction with two G-protein-coupled receptors, B1R and B2R. Most responses to BK depend on B2R activation, which involves the activation of the phospholipase C (PLC) or cyclic adenylate cyclase (AC)/AMP (cAMP) signalling pathway, promoting the subsequent activation of PKC or PKA, respectively [74]. Significantly, the B1R receptor is not expressed under normal physiological conditions, but its expression increases in response to inflammation and tissue damage [75]. The role of BK in the nociceptive response might depend on TRPV1 activation, as demonstrated by the significant increase in TRPV1 reactivity and protein mRNA expression observed in primary rat nociceptive sensory neurons cultured in the presence of PGE2 and BK [76]. Furthermore, BK 2qacan modulate TRPA1 activity. Indeed, TRPA1 currents evoked by allyl isothiocyanate or cinnamaldehyde in human embryonic kidney cells (HEK293) expressing B2R were potentiated by the BK presence. This potentiating effect was not observed in the presence of a PLC inhibitor, suggesting the involvement of a PLC-dependent pathway in BK-induced sensitization and hyperalgesia [74].

### 4.2. Neuroplasticity and Sensitization

Although nociceptors are defined as receptors with a high activation threshold in the somatosensory nervous system, they are not static detectors as they can undergo plastic changes that lower their activation threshold [77]. Thus, nociceptors are subject to the phenomena of neuroplasticity, i.e., the ability to adapt to internal and external changes by modifying neuronal pathways and/or circuits. In the context of nociception, neuroplasticity involves the processing of somatic memories and functional changes of the nervous system as a reaction to injury [78].

Peripheral sensitization is a form of nociceptor plasticity evoked by chemical mediators released during inflammation or in the presence of tissue damage. Therefore, it is characterised by a lowering of the activation threshold of nociceptors, which are also stimulated by innocuous inputs that would not normally induce a nociceptive response [79]. In this regard, an important role is played by TRPV receptors, whose activity is based on the balance between phosphorylation and dephosphorylation, involving serine and threonine residues. Particularly, mediators released after tissue damage, by binding to their receptor, trigger the activation of specific kinases, such as PKA, PKC, PLC, or Ca^2+^-calmodulin-dependent kinase II (CAMKII), which phosphorylate TRPV receptors. Phosphorylation of these receptors is associated with sensitization, whereas dephosphorylation is associated with desensitization [78]. Sensitization of the TRPV1 receptor, whose function is the conduction of Na^+^, K^+^, and Ca^2+^ promoted by mediators of inflammation, depends not only on its phosphorylation, but also on increased membrane protein expression or the phosphatidylinositol 4,5-bisphosphate (PI(4,5)P2)-induced inhibition of TRPV1 [80,81]. This receptor is involved both in neuropathic pain and pain progression and chronicity. Interestingly, TRPV1 is known to form a heterodimer with TRPV2 in DRG neurons in some cases, although the function of this complex is not yet understood [82].

In contrast, central sensitization is a phenomenon of synaptic plasticity associated with a gain in function and response of neurons and circuits in nociceptive pathways, which occurs when painful stimulation is particularly intense or prolonged [83]. Although central sensitization was initially attributed to the persistence of peripheral noxious stimuli [84], this phenomenon is now known to depend on neuronal changes in the CNS unrelated to peripheral input [85]. Glutamate plays a key role in central sensitization development, as its release from primary nociceptive afferent terminals promotes the N-methyl-D-aspartate receptors (NMDARs) activation, which mediate Ca^2+^ influx by activating intracellular signalling that initiates and maintains central sensitization [85]. Alpha-amino-3-hydroxy-5-methyl-4-isoxazolopropionic acid receptors (AMPARs) are also involved in glutamate-mediated central sensitization. Indeed, peripheral inflammation has been reported to cause changes in AMPARs subunit trafficking in the dorsal horn, promoting the hypersensitivity underlying persistent pain [86]. Local application of glutamate by injection induces excitation and sensitization of nociceptors, suggesting its primary role in nociception and inflammation. This is particularly true in craniofacial musculoskeletal pain. Indeed, Chung et al. proposed a model in which the interaction between glutamate and its receptors leads to the activation of TRPV1 or TRPA1, resulting in the development and maintenance of craniofacial muscle hyperalgesia [87]. Specifically, in the proposed model, inflammation and muscle damage cause the release of chemical mediators that promote the activation of TRP ion channels, resulting in increased Ca^2+^ influx and glutamate release from afferent terminals. The released glutamate, acting in an autocrine or paracrine manner, activates glutamate receptors and PKC. PKC, in turn, phosphorylates TRPV1, which integrates signals from other ligands released following injury with those mediated by glutamate, promoting the development and maintenance of hyperalgesia [87]. However, although the model is novel and of great interest, further investigation is required as other mediators may be involved in the complex nociceptive glutamate signalling. Indeed, glutamate injection in mice causes an increase in the expression of various phosphorylated isoforms of PKA, which is known to be involved in nociception [88].

Central sensitization is a form of synaptic plasticity that characterises a variety of diseases of the musculoskeletal system and is among the main contributors to persistent pain in fibromyalgia, osteoarthritis, rheumatoid arthritis, and CRPS [89]. Therefore, research should not only be directed at understanding the molecular mechanisms that induce nociceptor stimulation, but a broader view is needed to better determine the role of central sensitization in chronic pain underlying musculoskeletal system diseases.

### 4.3. Nociceptive Neuropeptides

The release of chemical mediators following the noxious stimulus causes the activation of specific ion channels that generate a Ca^2+^ flux. This, in peripheral nerve terminals, induces the release of vesicles containing neuropeptides, including calcitonin gene-related peptide (CGRP), substance P (SP), and vasoactive intestinal peptide (VIP), which play several roles in the context of tissue damage and are known to induce or sensitize peripheral pain, especially in musculoskeletal disorders.

#### 4.3.1. Calcitonin Gene-Related Peptide (CGRP)

CGRP is a 37 amino acid peptide produced by the alternative RNA splicing of the calcitonin CALCA gene that promotes pain transmission and sensitization through interaction with a receptor heterodimer consisting of the calcitonin-like receptor (CRL) and receptor activity modifying protein 1 (RAMP1). In humans, two isoforms of this peptide have been identified, α-CGRP and β-CGRP, which differ in amino acid composition but have similar biological properties [90]. CGRP acts on smooth muscle cells and vascular endothelial cells, exerting a powerful vasodilatory action and contributing to the formation of tissue edema [91,92]. It also plays an important role as an inducer of angiogenesis and lymphangiogenesis [93].

In the musculoskeletal system, CGRP influences bone metabolism in several ways. For example, alpha-CGRP knockout is known to significantly influence bone resorption by reducing both the number of osteoclasts and basal mRNA levels of nuclear factor kappa-B ligand (RANKL) [94]. Furthermore, treatment of primary rabbit osteoblasts with human CGRP revealed increased staining for alkaline phosphatase (ALP) and reduced levels of RANKL, suggesting a role for this neuropeptide in regulating bone resorption by both promoting osteoblast differentiation and inhibiting osteoclast activity [95]. CGRP has been observed to promote bone formation and fracture healing. Primary afferent sensory neurons are CGRP-immunoreactive and are involved in the local control of bone formation, as they promote bone and cartilage mineralization [96]. Indeed, Zhang et al. found longer healing times in fractures combined with peripheral nerve injury, suggesting that CGRP-positive fibres influence the optimal maintenance of bone integrity and fracture healing [97].

In 2017, Schou and colleagues studied the association between CGRP and pain of various types, including pain in musculoskeletal structures [98]. In patients with knee osteoarthritis, CGRP levels in serum and synovial fluid were significantly higher than in controls, and this increase was correlated with pain intensity. Similarly, high levels of CGRP were also found in the hip synovium of osteoarthritic patients and in the synovial tissue of patients with temporomandibular joint (TMJ) pain. The positive correlation between pain and CGRP levels was also observed in patients with shoulder and neck pain and in patients with meniscal or ligamentous injuries, confirming a neuromodulatory role for CGRP in central and peripheral nociceptive pathways [98].

#### 4.3.2. Substance P (SP)

SP is an 11 amino acid peptide that co-localises with CGRP in primary afferent sensory fibres and promotes nociceptive signalling through the activation of neurokinin receptor 1 (NK1R). In addition, like CGRP, it has a potent vasodilatory action [99]. NK1R receptors are expressed by both osteoblasts and osteoclast precursors, suggesting a role for SP in bone metabolism. Indeed, the treatment of bone marrow stem cells (BMSCs) with SP is known to induce cell proliferation as well as ALP and osteocalcin (OCN) expression, confirming the involvement of the neuropeptide in osteoblastic differentiation. Furthermore, the treatment of bone marrow macrophages (BMM) with SP, in the presence of RANKL and colony-stimulating factor, significantly promoted osteoclastogenesis through the nuclear translocation of NF-kB [100].

SP is also an important regulator of angiogenesis, with a key role in fracture healing. In this regard, Niedermair et al. used an SP-deficient mouse model (Tac-/-) to determine the effect of SP deficiency on fracture healing [101]. At day 13 after fracture, a reduction in the area covered by hypertrophic chondrocytes was found in Tac-/- mice, highlighting a potential role of SP in cartilage ossification during fracture healing. SP-immunoreactive fibres reached maximum density approximately two weeks after fracture, and then returned to basal levels within 25 days, suggesting that SP release from sensory neurons could promote fracture healing and regulate bone turnover [101].

The SP expression in DRG neurons and in the dorsal horn, but not in the ventral horn, is evidence of its involvement in primary sensory afferent neurotransmission. Interestingly, several primary afferent fibres express both SP and glutamate, which are known to lower the pain threshold of dorsal horn projection neurons after their release. SP release might enhance glutamate signalling in these neurons because interaction with NK1R induces PLC activation, facilitating glutamatergic transmission through phosphorylation of NMDA receptor subunits. Indeed, through patch-clamp recordings on rat dorsal horn neurons, glutamate-induced currents were observed to undergo potentiation in the presence of SP, reinforcing the hypothesis of its involvement in nociception and sensitization [99]. In this context, Lisowska et al. evaluated the correlation between chronic pain and SP levels in seventy patients with osteoarthritis and rheumatoid arthritis and found an elevated serum concentration of SP in all of them, confirming the correlation between the neuropeptide, chronic inflammation, and chronic pain [102].

#### 4.3.3. Vasoactive Intestinal Peptide (VIP)

VIP is a 28 amino acid peptide produced by the cleavage of the pre-pro-VIP precursor, which interacts with two G-protein-coupled receptors, VPAC1 and VPAC2. Furthermore, a third receptor, PAC1, can recognise VIP with a very low affinity. In addition to its vasodilatory action, VIP plays multiple roles in both physiological and pathological contexts in the development, growth, and control of neuronal, epithelial, and endocrine functions [103].

Conflicting results have been reported regarding the effect of VIP on bone metabolism. However, recent evidence has shown the ability of VIP to induce osteogenic differentiation of BMSCs and to significantly improve repair in a rat model of cranial bone defect by increasing both bone formation and angiogenesis [104]. Furthermore, the presence of VIP receptors on osteoblasts and osteoclasts suggests that this neuropeptide may influence bone metabolism by regulating the function or activity of both cell types [105]. Interestingly, bone tissue is abundantly innervated by VIP-positive sensory and sympathetic nerve fibres, suggesting the involvement of this neuropeptide in fracture healing. In this regard, Shi and colleagues studied the effect of VIP on femoral fracture in a mouse model undergoing chemical sympathectomy and found a significant improvement in fracture healing in terms of both bone mineralisation and mechanical properties after VIP treatment, indicating that VIP-induced vasodilation of bone arteries may be crucial in the healing process [106].

Finally, VIP may in some way contribute to osteoarthritis progression and pain development in this disease, although this relationship is not entirely clear. Particularly, synovial fluid and articular cartilage in patients with knee osteoarthritis are known to show reduced expression of VIP which would contribute to increased joint inflammation, with increased expression of pro-inflammatory cytokines and PGE2 and cartilage degradation. However, it has also been suggested that, in arthritic knees, the up-regulation of VIP increases the production of IL-6, IL-1β, and TNF-α, increasing sensitization of afferent nerve terminals and inducing pain [107]. Kanemitsu et al. recently showed a positive correlation between VIP expression in subchondral bone and osteoarthritis progression, indicating the peptide’s involvement in joint pathology and suggesting the potential use of antagonists of VIP signalling to counteract the pathology [108].

## 5. Chronic Pain in Musculoskeletal Disorders

Musculoskeletal disorders share a common factor in chronic pain, which has a significant impact on patients’ quality of life and functional status. Although the musculoskeletal pain that characterises these disorders can be distinguished into primary and secondary, some evidence suggests the involvement of neuropeptides, neurotransmitters, and other mediators in the development and maintenance of pain and subsequent central sensitization. Below, we discuss for representative purposes some disorders associated with chronic musculoskeletal pain.

### 5.1. Osteoarthritis

Osteoarthritis is an age-related pathological condition characterised by a progressive deterioration of hyaline articular cartilage, subchondral bone, ligaments, as well as capsule and periarticular muscles, resulting in joint destruction [109,110]. In several high-income countries, the cost of medical expenses associated with osteoarthritis has been estimated to be 1–2.5% of gross domestic product, underlining the high economic burden associated with this disease [111]. In addition, this burden is likely to increase as the global prevalence of osteoarthritis is rising due to both the continued aging of the population and the increased incidence of the major risk factor associated with this disease, obesity [112].

Inflammatory pain is the main symptom of osteoarthritis, and, in this context, a central role is played by the crosstalk between the immune system and nociceptive neurons [113]. However, cartilage is an aneurysmal tissue and cannot directly generate pain. In contrast, other tissues that constitute the joint, such as the subchondral bone, periosteum, and joint capsule, are richly innervated by nociceptive A and C fibres and could be responsible for pain perception [114]. Thus, immune cells, as well as mesenchymal, glial, and epithelial cells, may release a wide variety of inflammatory mediators responsible for nociceptive stimulation and, consequently, pain sensation. Nociceptive neurons, in turn, release the neuropeptides CGRP and SP that promote vasodilation and extravasation, drawing immune cells to the site of inflammation [113]. Not surprisingly, such stimulation of nociceptive neurons leads to a state of peripheral sensitization, manifested by mechanosensitivity, i.e., a lowering of the pain threshold induced by mechanical stress [115].

In addition to the mechanisms of peripheral nociceptive pain due to inflammation and tissue damage, central sensitization may also be involved in the development of osteoarthritic pain [116,117,118]. In this regard, an important role has been suggested for NGF, as demonstrated by the increase in its expression and release induced by both mechanical and inflammatory stress observed in murine cartilage explants in vitro [119]. The role of NGF in the pain development has also been demonstrated by studies evaluating the efficacy of using anti-NGF antibodies (tanezumab) for the treatment of knee and hip osteoarthritis, suggesting that NGF signalling inhibition, involving activation of TrkA and p75NTR receptors, may represent a viable pain management strategy in osteoarthritic patients [120]. However, some evidence has reported the occurrence of adverse events after treatment with high-dose tanezumab in combination with non-steroidal anti-inflammatory drugs (NSAIDs), including osteonecrosis and rapid progression of knee, hip, and shoulder osteoarthritis, highlighting the need for further understanding of the molecular mechanisms involved in the osteoarthritic pain development [121].

Undoubtedly, osteoarthritis pain has a complex pathophysiology, with both peripheral and central manifestations, highlighting the need to treat the algic condition not only as a part of the osteoarthritis symptomatology, but as a well-defined disease that can seriously compromise the patient’s quality of life, regardless of the degenerative disease process.

### 5.2. Osteoporosis

Osteoporosis is a musculoskeletal disorder characterised by the progressive reduction of bone mass and the deterioration of tissue microstructure, leading to an increase in fragility fractures caused by an increased risk of falls [122]. This pathology is enormously prevalent among the elderly and post-menopausal women, representing a major economic burden on health care systems and a major cause of disability and mortality [123]. Among the various hallmarks of osteoporosis, bone pain and chronic back pain due to multiple vertebral fractures is undoubtedly one of the most disabling [124]. In the context of musculoskeletal pain due to osteoporosis, an important role has been suggested for IL-6, as treatment with antibodies to IL-6R is known to reduce mechanical hyperalgesia in the hind limbs of ovariectomized rats. Interestingly, a reduced number of DRG CGRP-positive neurons was also observed, suggesting IL-6 as a major contributor to post-menopausal osteoporotic pain [125]. Worth noting is that the same authors subsequently evaluated the efficacy of anti-IL-6R antibody in a mouse model of limb unloading, observing again, a reduction in both mechanical hyperalgesia and CGRP expression in DRG neurons [126]. Although these results support a crucial role for IL-6 signalling in the development of osteoporotic pain and subsequent sensitization, the mechanisms underlying this phenomenon are far more complex and not yet fully understood. In this regard, Zheng et al. studied the effect of an SP receptor antagonist on hyperalgesia and bone metabolism in ovariectomized mice and found a significant increase in the nociceptive threshold, suggesting a dependence of estrogen deficiency-induced hyperalgesia on SP up-regulation and its receptor expression [127].

Further observations were provided by Xiao and colleagues, who evaluated the distribution of CGRP, SP, VIP, and Neuropeptide Y (NPY) in association with joint pain in femoral head spongiosum bone biopsies taken from 10 women with osteoarthritis and 10 women with osteoporosis during hip arthroplasty surgery. The mean optical density (MOD) values obtained by immunohistochemical analysis were correlated with pain, measured by the VAS scale before surgery. Interestingly, the VAS score correlated positively with MOD values of CGRP, SP, and VIP, and negatively with MOD values of NPY in all patients, although a significantly higher expression was observed in patients with osteoarthritis [128].

Taken together, this evidence confirms the involvement of neuropeptides in the genesis and maintenance of osteoporosis pain, although knowledge regarding the specific mechanisms that determine central sensitization is still limited. Investigating the role of these substances in the pathogenesis of osteoporosis and the development of related pain is essential for the adoption of strategies to minimise the impact of this disabling symptom in osteoporotic patients.

### 5.3. CPRS-1

CRPS-1 is a disorder characterised by physiological changes not related to an injury to neural tissue, which leads to pain onset and promotes disability [129]. This condition arises following a minor trauma, such as a bone fracture or sprain, and manifests itself through symptomatology out of proportion to the triggering event, particularly with the phenomena of allodynia and hyperalgesia [130]. Again, the inflammatory process leads to the production of cytokines and other chemical mediators that promote, in addition to peripheral sensitization, the release by primary afferent neurons of CGRP and SP, which are responsible for redness, edema, and skin hyperemia, changes that are collectively labelled as neurogenic inflammation [131]. Furthermore, these neuropeptides have been shown to be primarily responsible for sensitizing second-order neurons in the spinal cord, promoting the participation of a central component in the pain development by CRPS-1 [132]. Thus, the excessive release of cytokines and neuropeptides promotes altered glutamate release in the CNS and aberrant activation of the NMDA receptor. The resulting deregulation of Ca^2+^ flux leads to increased synaptic strength and function, which results in increased spontaneous pain and reaction to peripheral stimuli [89].

The involvement of local inflammation in the numerical increase of NMDA receptors in peripheral tissue and sensory nerves has also been reported, further contributing to the sensitization process [133]. Not surprisingly, some of the strategies used in CRPS-1 pain management are NMDA receptor antagonists. Indeed, the intravenous administration of ketamine for several days is known to induce significant pain reduction in patients with CRPS-1 [134], although its use has been associated with several side effects, such as tachycardia, hallucinations, hypertension, and severe drug high [135]. In this regard, new therapeutic approaches to counteract the hyperalgesia and allodynia typical of CRPS-1 have been developed, including bisphosphonates [136,137]. Finally, a key role has been suggested for TRPA1, as demonstrated by the significant reduction in mechanical allodynia observed in rats with ischemia/reperfusion-induced CRPS-1 after treatment with TRPA1 receptor antagonists [138].

Overall, CRPS-1 pathophysiology is extremely complex, being characterised by local inflammation, vasomotor changes, and peripheral and central sensitization. However, the mechanisms responsible for the maintenance of pain remain unexplored, highlighting the need for further experimental studies to better understand pain chronicity.

### 5.4. Fibromyalgia

Fibromyalgia is a syndrome characterised by chronic musculoskeletal pain, muscle and joint stiffness, fatigue, depression, and cognitive dysfunction, which affects approximately 5% of the population with a higher incidence in women than men [139].

Although much of the research regarding the mechanisms underlying fibromyalgia is directed at CNS involvement, a role for immune cells, such as mast cells, monocytes, and neutrophils, has also been suggested in the pathogenesis of this condition [140,141]. In some studies conducted on the plasma of patients with fibromyalgia, an increase in pro-inflammatory cytokines, such as IL-6, interleukin-8 (IL-8), IL-1β, and TNF-α, was found [142,143]. However, inflammation may be only one of the components responsible for fibromyalgia pathogenesis, in which neuroendocrine, genetic, oxidative, and environmental factors are also involved [139]. Further confirming the role of inflammation in fibromyalgia pathogenesis and associated pain, Tsilioni et al. found increased SP and haemokinetic-1 (HK-1), structurally related to SP, as well as increased levels of IL-6 and TNF-α in the serum of fibromyalgia patients, suggesting mast cells as the main mediators of inflammation in fibromyalgia [144]. However, despite the important role of SP and the NK-1 receptor in pain pathophysiology, the NK-1 blockade is not known to improve pain in fibromyalgia, highlighting its criticality in pain transmission [145]. Finally, CGRP may also be involved in fibromyalgia pain, as demonstrated by the significantly higher serum levels found in fibromyalgia patients compared to control subjects [146].

Despite the participation of inflammatory mediators in the development of pain symptoms, the main feature of fibromyalgia remains the central sensitization phenomenon responsible for hyperalgesia. Indeed, studies in human patients and animal models have shown that the blockade of NMDAR enhances hyperalgesia, suggesting sustained glutamatergic activity underlying the sensitization [147,148]. Unfortunately, as observed in studies for CRPS-1, NMDAR antagonists, such as ketamine or memantine, are associated with a wide spectrum of side effects, which severely limits their clinical use [149,150].

Therefore, fibromyalgia is a multifactorial disorder in which there is neither obvious nociceptor activation nor a proven nerve injury, so that the pain associated with this disorder has been defined by the new term “nociplastic” [1]. Further studies are needed to determine the contribution of mediators released by immune cells to the pain symptoms of this condition.

## 6. Musculoskeletal Pain Management: Approaches and Perspectives

Although there are precise therapies for musculoskeletal pain treatment, it is important to remember that pain should not be considered simply as a symptom of a particular pathology, but rather as a well-defined condition requiring rapid and effective intervention. For this reason, intervening in the algic condition is crucial to improve the patient’s clinical, emotional, and psychological state. Not surprisingly, the management of chronic pain is very complex, as its outcome is related to the emotional state of the patient [151].

The NSAIDs administration is generally the first line of defence against musculoskeletal pain. Although the efficacy of this class of drugs in improving pain has been widely demonstrated, the beneficial effects are only apparent for short periods, and are, therefore, not a long-term treatment strategy [152]. Similar results have been observed for other classes of drugs such as cyclooxygenase inhibitors and opioids, with a greater number of adverse events, including opioid-induced hyperalgesia, that advise against their long-term use. Corticosteroid injections are also not a valid treatment strategy, as their long-term efficacy has been compared to that of non-pharmacological interventions, such as exercise [152]. Overall, pharmacological therapy alone appears to be insufficient for musculoskeletal pain management, suggesting the need for a multidisciplinary approach whereby professionals from different disciplines work together with the common goal of developing a multimodal treatment that includes both pharmacological and non-pharmacological approaches. In this regard, several practices have been adopted to improve the algic condition, such as transcutaneous electrical nerve stimulation (TENS), ultrasound, and acupuncture. However, the efficacy of these strategies in musculoskeletal pain management is supported by limited or low-quality evidence, providing no proof of their clinical effectiveness in improving pain [153,154,155]. Interestingly, exercise has been shown to promote significant improvements in the context of chronic musculoskeletal pain, although it is not yet universally accepted which type of exercise offers the greatest effectiveness [156]. In this regard, Busch and colleagues summarised in a systematic review the results of 34 RCTs evaluating the effectiveness of aerobic training and muscle strengthening on the well-being of individuals with fibromyalgia. Of note is that both types of training have been reported to exert beneficial effects on physical function, improving pain status and general well-being in patients with this condition [157]. Similarly, Bartels et al. evaluated the effect of aquatic exercise in people with knee and/or hip osteoarthritis and reported that this type of exercise has clinically relevant results for pain, disability, and quality of life in people with osteoarthritis [158].

Finally, education in pain neuroscience represents another important type of intervention used in chronic musculoskeletal pain management, with the aim of reconceptualising knowledge about pain and educating the patient about the elaboration and sensitization processes. Importantly, recent evidence has shown that this approach can significantly help patients to cope with their pain condition, suggesting that the effectiveness of pharmacological and non-pharmacological therapies aimed at pain management should directly involve the patient with an educational approach. In fact, knowledge of the pain neurophysiology makes patients aware of their condition, reducing the risk of adopting incorrect behaviours such as kinesiophobia, sedentariness, and depression [159,160].

**Figure 3:**
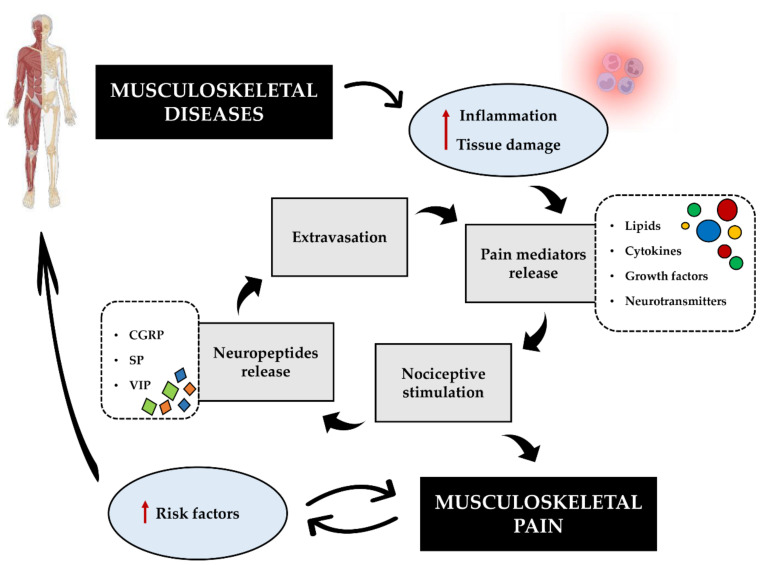
Musculoskeletal pain cycle. Some musculoskeletal disorders are characterised by an increased inflammatory state and tissue damage, which leads to the release of chemical mediators responsible for the nociceptive response. Consequently, the release of neuropeptides such as calcitonin gene-related peptide (CGRP), substance P (SP), and vasoactive intestinal peptide (VIP) occurs, with a potent vasodilatory action that promotes the extravasation of immune cells to the site of damage. In a pathological context, this chain of events takes on the characteristics of a vicious circle leading to prolonged nociceptive stimulation. The resulting musculoskeletal pain leads to the exacerbation of important risk factors, such as sedentariness and depression, which not only promote musculoskeletal pain but also exacerbate the pathological state by promoting the reinforcement of the cycle.

In this regard, we propose the establishment of a musculoskeletal pain cycle, which, if not interrupted in time, leads to central sensitization and chronicity (Figure 3). Indeed, several mediators released by immune cells during inflammation or due to tissue damage are known to be responsible for nociceptive stimulation and peripheral pain. This results in the release of certain neuropeptides from peripheral nerve terminals, which are actively involved in the development and maintenance of pain. In addition, the vasodilatory action of these neuropeptides promotes the extravasation of immune cells at the site of damage, giving them an essential role in reparative processes as well.

However, in a pathological context, the persistence of the noxious stimulus leads to prolonged nociceptive stimulation, increasing the risk of chronicity and contributing to the exacerbation of some important risk factors, such as sedentariness and depression. These may lead to poor patient cooperation in treatment, worsening both pain and disease. Crucial, in our view, is the self-perpetuating nature of this cycle, which underlines the importance of the rapid and appropriate management of primary or secondary chronic musculoskeletal pain due to prolonged or impaired nociceptor stimulation. Finally, we suggest that the patient needs to understand the real risks of misguided behaviour and inappropriate approach to pain, so that the treatments used to manage pain are effective. Overall, although our model cannot explain the clinical and molecular complexity of musculoskeletal pain, we believe that it can make a useful contribution to patient motivation, with the aim of promoting full cooperation and discouraging the adoption of self-harming behaviour.

## 7. Conclusions

**Figure 4:**
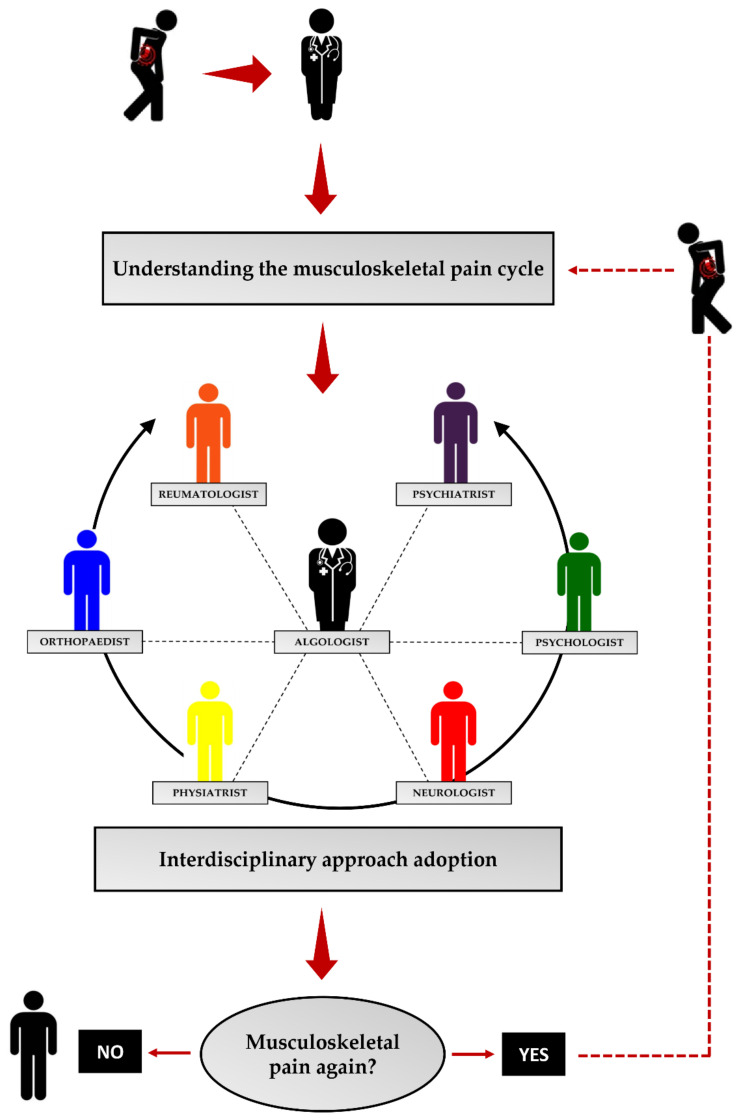
Possible management algorithm for the patient with musculoskeletal pain. An understanding of the musculoskeletal pain cycle may be the starting point for the development of an appropriate management algorithm for the musculoskeletal pain patient. If the patient understands the risks of a superficial and inconsistent approach, he/she will be more cooperative in accepting the therapy provided by a multidisciplinary team. Particularly, an algologist or pain specialist should play the role of team leader of a specialist team, such as an orthopaedist or bone specialist, a physiatrist or specialist in physical and rehabilitation medicine, a neurologist or nerve specialist, a psychologist and a psychiatrist or mental health specialists, and a rheumatologist or specialist of joints and musculoskeletal system. In addition, if therapy proves ineffective, knowledge of the pain cycle may reduce the likelihood of counterproductive and self-defeating behaviour, promoting greater adherence to therapy.

Musculoskeletal pain has an extremely negative impact on all aspects of an individual’s life. This condition is increasingly prevalent in the population, highlighting the ineffectiveness of currently available approaches and the consequent need to develop management algorithms that can facilitate the patient in the complex therapeutic pathway. Unfortunately, today, many hospitals still do not have a pain management centre capable of providing satisfactory patient care. Such centres should provide an interdisciplinary approach to pain management, i.e., multiple specialists should co-operate to produce the best possible treatment. However, often the collaboration between these specialists is not as strong as it should be, generating confusion in the patient and increasing the risk of resignation to the pain condition. In fact, to date, only a small proportion of patients with musculoskeletal pain receive adequate care at specialised pain management centres, underlining both the need to increase the means available to combat musculoskeletal pain and the duty to educate the patient about the risks associated with neglected pain. In this regard, it is our opinion that the first step in defeating the enemy called pain is for the patient to be fully aware of the mechanisms that promote its maintenance, to avoid adopting an inappropriate attitude to the problem and, consequently, resignation to musculoskeletal pain (Figure 4). Knowledge of the molecular mechanisms underlying the development and persistence of pain could help to raise patient awareness and promote full adherence to treatment. In this context, the musculoskeletal pain cycle we propose could represent the starting point for the development of a treatment algorithm for the patient with pain. Indeed, if the patient understands the risks associated with the underestimation of pain, he/she may be more committed to the treatment pathway, increasing the probability of a positive outcome. On the contrary, knowledge of the physiopathological mechanisms and the conscious risk of chronicity could prevent resignation to the algic condition, favouring the continuity of therapy.

Although it may seem to be a limiting factor, the simplicity of the musculoskeletal pain cycle is its strength, making it easy to understand. Furthermore, understanding it does not require a high level of education, making it an accessible tool for all patients. Thus, a simple model could be a crucial ally in understanding and defeating a devious and insidious enemy: musculoskeletal pain.

## Data Availability

Not applicable.
